# Determination of Urinary Cotinine Cut-Off Concentrations for Pregnant Women in the Japan Environment and Children’s Study (JECS)

**DOI:** 10.3390/ijerph17155537

**Published:** 2020-07-31

**Authors:** Yukiko Nishihama, Shoji F. Nakayama, Takahiro Tabuchi, Tomohiko Isobe, Chau-Ren Jung, Miyuki Iwai-Shimada, Yayoi Kobayashi, Takehiro Michikawa, Makiko Sekiyama, Yu Taniguchi, Hiroshi Nitta, Shin Yamazaki, on behalf of the Japan Environment and Children’s Study Group

**Affiliations:** 1 Japan Environment and Children’s Study Programme Office, Centre for Health and Environmental Risk Research, National Institute for Environmental Studies, Tsukuba, Ibaraki 305-0053, Japan; nishihama.yukiko@nies.go.jp (Y.N.); isobe.tomohiko@nies.go.jp (T.I.); jung.chau-ren@nies.go.jp (C.-R.J.); iwai.miyuki@nies.go.jp (M.I.-S.); kobayashi.yayoi@nies.go.jp (Y.K.); takehiro.michikawa@med.toho-u.ac.jp (T.M.); sekiyama.makiko@nies.go.jp (M.S.); taniguchi.yu@nies.go.jp (Y.T.); nitta@nies.go.jp (H.N.); yamazaki.shin@nies.go.jp (S.Y.); 2 Cancer Control Center, Osaka International Cancer Institute, Osaka 541-8567, Japan; tabuti-ta@mc.pref.osaka.jp; 3 Department of Environmental and Occupational Health, School of Medicine, Toho University, Tokyo 143-8540, Japan

**Keywords:** cotinine, pregnant women, cut-off value

## Abstract

Few studies have assessed the accuracy of self-reported questionnaires to determine smoking habits relative to urinary biomarkers. This study investigated urinary cotinine cut-off concentrations distinguishing active, passive and non-smokers among pregnant women who participated in the Japan Environment and Children’s Study, a nationwide birth cohort study. Pregnant participants with measured urinary cotinine concentrations (UCCs) and who completed self-reported questionnaires on smoking status were included (*n* = 89,895). The cut-off values (COVs) for active and passive smokers were calculated by fitting mixed normal distribution functions to UCCs. The sensitivity and specificity of the questionnaires were subsequently evaluated. The median (interquartile range) UCC was 0.24 (0.083–0.96) µg/g-creatinine, with the detection rate of 89%. The COV for distinguishing active smokers from passive and non-smokers was 36.8 µg/g-creatinine. When this COV was considered to represent the true condition, the questionnaire had a sensitivity of 0.523, a specificity of 0.998, a positive predictive value (PPV) of 0.967 and a negative predictive value (NPV) of 0.957. The COV for distinguishing passive smokers from non-smokers was 0.31 µg/g-creatinine, with the questionnaire having a sensitivity of 0.222, a specificity of 0.977, a PPV of 0.868 and an NPV of 0.644. As many as 78% of passive smokers might be misclassified as non-smokers.

## 1. Introduction

Maternal smoking and/or exposure to environmental tobacco smoke (ETS) have been found to have adverse effects on the health and development of foetuses and children, including higher foetal heart rate, lower birth weight, altered neurobehaviour and asthma [1,2,3,4]. Maternal smoking status is regarded as an important covariate in birth cohort studies testing the effects of maternal environmental exposure on child health [5,6].

The Japan Environment and Children’s Study (JECS) is an ongoing nationwide birth cohort study started in 2011. JECS was designed to investigate the effects of environmental factors on child health and development [7,8]. A total of 103,099 pregnant mothers were registered, with all of their babies born by 2014. Biospecimens, including blood, urine, hair and breast milk, were collected from mothers during pregnancy, at delivery and/or one month after birth. Cord blood and child hair samples were also collected.

The cigarette smoking rate among Japanese women has not changed in the last decade, being approximately 8%, according to the National Health and Nutrition Survey [9]. Of the pregnant women who participated in JECS and completed the self-administered questionnaire, 5.4% reported smoking during pregnancy [10], compared with 7.2% of pregnant women in the United States in 2016 [11]. Despite knowledge about the hazards of tobacco smoking during pregnancy [3,12], more than 15% of the mothers who participated in JECS reported that family members smoked in their homes [8].

Cotinine is a metabolite of nicotine, and serum or urinary cotinine concentration is regarded as a biomarker for smoking [13]. Most studies investigating serum/urinary cotinine concentrations as markers for smokers and ETS exposure used self-reported questionnaires to determine the actual smoking condition [14,15,16]. Although several studies reported the accuracy of the self-reporting questionnaire for the evaluation of smoking status [14,17,18], their results were inconsistent, with sensitivities ranging from 82% to 100% and specificities from 81% to 97%. A cut-off value (COV) is often determined by comparing a test method of interest against a gold-standard method [19]. The commonly used technique is receiver operating characteristic (ROC) curve analysis; however, it assumes the existence of a gold-standard test to determine the true conditions. Habibzadeh et al. also suggested an analytical method to derive a COV for continuous results. This also assumes there is a way to determine the true conditions. Almost all previous studies that proposed COVs for urinary cotinine concentrations used ROC curve analysis and considered questionnaire results as a gold-standard [14,16,17]. To the best of our knowledge, only one study to date used plasma cotinine concentrations as a gold-standard test and estimated the sensitivity and specificity of self-reported questionnaires for smoking status [20]. Du et al. suggested the use of the expectation–maximization (EM) algorithm to find the best fit mixture model and then derivation of a COV from the mixture model when a gold-standard test is unavailable [21]. This method employs the EM algorithm to fit multiple univariate empirical distribution functions to continuous measurement data. Then, a COV is calculated as the point that maximizes the sum of sensitivity and specificity, i.e., by minimizing the number of false positive and false negative cases. The questionnaire on smoking status is less accurate [13]. Therefore, in this study, we considered urinary cotinine as a gold-standard and used Du et al.’s method to estimate COVs.

The primary aim of JECS was to evaluate the effect of exposure to the environment, especially chemical substances, on child health. Exposure to tobacco smoke should become a major covariate as well as an important exposure for all analyses within JECS. The present study was primarily performed to investigate cut-off urinary cotinine concentrations indicative of pregnant mothers’ smoking status. The second aim of this study was to evaluate the comparative accuracy of the self-reported questionnaire when the urinary cotinine concentrations were considered to represent the true condition.

## 2. Materials and Methods 

### 2.1. Study Participants

The JECS protocol is described in detail elsewhere [7,8]. Briefly, JECS is an ongoing nationwide birth cohort study that registered over 100,000 pregnant women from January 2011 to March 2014 in 15 study areas across Japan. JECS is funded by the Ministry of the Environment of Japan and operated by the National Institute for Environment Studies in coordination with the National Center for Child Health and Development and 15 regional centres. Written informed consent was obtained from all participating women and their families. The current study was based on the jecs-ta-20190930 dataset (*n* = 104,062), which was released in September 2019. Women with multiple birth pregnancies (*n* = 1002), women lacking measurements of urinary cotinine concentration (*n* = 7043), women registered at the incorrect gestational week (*n* = 563) and those whose urine samples were collected more than 60 days before or after their responses to the mid-late pregnancy questionnaire were received (*n* = 5405), were excluded from analysis. Participants who did not report their own smoking status or the smoking status of their partners on the questionnaires (*n* = 154) were excluded for determination of urinary cotinine cut-off concentrations, and those who did not report the state of passive smoking (*n* = 758) and were active smokers (*n* = 4034) were also excluded from analyses of passive smoking using questionnaires (leaving *n* = 85,103, see Figure S1).

### 2.2. Sample Collection

A maternal urine sample was collected at the second or third trimester of pregnancy using a 120 mL polypropylene container (VWR International, LLC., Wayne, PA, USA). A 15 mL aliquot of each urine sample was placed into three 5 mL Data Matrix code-labelled cryogenic biobanking tubes (Greiner Bio-One International GmbH, Kremsmünster, Austria), transferred to a contract laboratory at 1–10 °C and stored at −80 °C until analysis. In the present study, 100 µL aliquots of urine were used to determine cotinine concentrations.

### 2.3. Data Collection

Urinary cotinine concentrations as well as urinary specific gravity and creatinine concentrations were determined by the method and verified by the quality control procedure described in Appendix A. The self-administered questionnaires included demographic, socioeconomic, lifestyle and health related information. Participants were asked to complete two questionnaires during pregnancy, one at enrolment during the first or second trimester (M-T1) and the other during the second or third trimester (M-T2). Smoking status of each mother and partner was scored on the questionnaires as never smoked, quit before the current pregnancy, quit after recognizing the current pregnancy or smoked during the current pregnancy. The questionnaires also included questions about the number of cigarettes smoked per day and passive smoking opportunities, which were scored as never exposed to passive smoking, and exposed to passive smoking for >1, >2, >4 and 7 days per week. Data on smoking status were based primarily on the M-T2 questionnaire, with results from the M-T1 questionnaire used to complement maternal smoking status when it was missing from the M-T2 questionnaire (the rate of complementation was 0.7%). Annual household income was reported as <4 million JPY (~36,844 USD), 4 to <6 million JPY (~55,266 USD) or ≥6 million JPY. Education was defined as <12 years or ≥13 years, as reported on the M-T2 questionnaire. Consumption of foods that were potential sources of nicotine, such as nightshades (bell peppers, aubergines, tomatoes and potatoes) and tea leaves (tea, Japanese tea and oolong tea) [22,23], was estimated using a food frequency questionnaire (FFQ), which was administered at the same time as the M-T2 questionnaires [24]. Maternal age at childbirth was determined from individual medical records, the maternal consent form and prenatal care records.

### 2.4. Data Analysis

**Figure 1:**
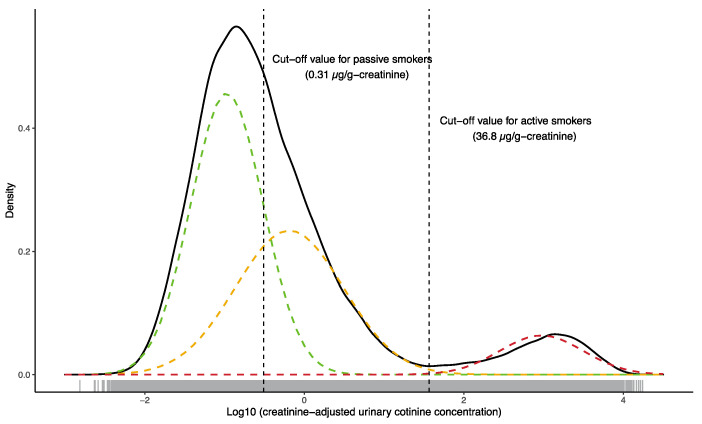
Distribution of urinary cotinine concentrations in active smokers, passive smokers and non-smokers. The solid line illustrates the probability density plot of the logarithm of creatinine-adjusted urinary cotinine concentrations to the base 10. Dashed lines represent the normal distributions fitted to the original data using a nonparametric EM-like algorithm. Dashed vertical lines show urinary cotinine cut-off concentrations. A rug plot is depicted on the x-axis.

**Figure 2:**
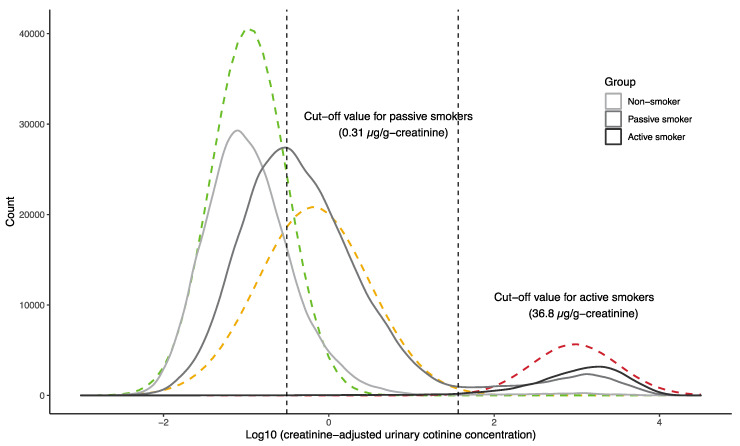
Smoothed histogram of urinary cotinine concentrations grouped by questionnaire responses. Groups of current smokers, passive smokers and non-smokers determined with the questionnaire are represented by the dark grey, grey and light grey solid lines, respectively. The fitted normal distributions are represented by the dashed lines. Dashed vertical lines show urinary cotinine cut-off concentrations.

Urinary cotinine concentrations normalized relative to creatinine concentrations were log10-transformed for statistical analysis. The association between consumption of foods that might be a potential source of nicotine and urinary cotinine concentration was evaluated by Spearman’s rank correlation coefficient, with no association found (see Figure S2), indicating that foods were a negligible source of urinary cotinine in subsequent analyses. Du et al.’s method [21] was employed to derive COVs for urinary cotinine concentrations distinguishing active smokers from others and passive smokers from non-smokers. Urinary cotinine concentrations were first log10-transformed and the EM-like algorithm was applied (R package ‘mixtools, ver. 1.2.0’ was used) to fit multiple univariate normal distribution functions. The goodness of fit of the different numbers of distributions (two, three and four) was evaluated using the Kolmogorov-Smirnov test, with a mixture of three normal distributions resulting in the best fit. Approximately 11% of the urinary cotinine concentrations were below the minimum reporting level (MRL). Although substitution of values below the MRL by one-half of the reporting limit has been used frequently in related studies, this type of approach may introduce an invasive pattern (signal) into the original data [25]. Urinary cotinine concentrations below the MRL were therefore imputed by randomly assigning values generated by the fitted mixture distribution function (Figure 1). We assumed that the distribution with the lowest mean represented for non-smokers (l-probability density function or l-PDF), the middle distribution represented passive smokers (m-PDF) and the highest distribution included active smokers (h-PDF). Then, the COV that maximized the sum of the sensitivity or true positive (area under h-PDF to the right of the COV) and the specificity or true negative (area under m-PDF to the left of the COV) was determined and assigned as the COV to distinguish active smokers from passive/non-smokers (Figure 2). The COV that distinguished passive smokers and non-smokers was derived in the same manner using l-PDF and m-PDF. Bootstrapping with 500 iterations was used to derive the 95% confidential interval estimates of each COV. Finally, the sensitivity and specificity of the questionnaire were evaluated using urinary cotinine cut-off concentrations as the true conditions. The COVs for unadjusted urinary cotinine data were calculated using the same method as described above.

The smoking status of each mother and partner obtained from the questionnaire was categorized as ‘active smoker (smoked during the current pregnancy),’ ‘passive smoker’ or ‘non-smoker (never smoked, quit before the current pregnancy, quit after recognizing the current pregnancy and did not have second-hand smoke exposure).’ Information about passive smoking was also obtained from the questionnaire, as experiencing ETS exposure for 7, 4–6, 2–3 or 1 day(s) per week. The smoking status of each partner was categorized as a current smoker or current non-smoker. The accuracy of the questionnaires was evaluated by urinary cotinine concentrations. The goodness of fit of the distribution functions for each smoking status was evaluated using the Kolmogorov-Smirnov test. Lastly, simple linear regression analysis was performed to examine the relationship between the average number of cigarettes smoked per day and average urinary cotinine concentrations. All statistical analyses were performed using R version 3.6.2 [26].

## 3. Results

### 3.1. Method Performance

The MRL of cotinine was 0.03 ng/mL. The reproducibility and intermediate precision for cotinine analysis were 4.0% and 4.7%, respectively.

### 3.2. Concentrations of Cotinine in Maternal Urine Samples

The demographic characteristics of the study participants are summarized in Table 1. According to their responses to questionnaires, 4.6% of pregnant women and 46.8% of their partners smoked during pregnancy. Cotinine was detected in 89% of the maternal urine samples, with the median (interquartile range (IQR)) concentration normalized to creatinine after imputation being 0.24 (0.083–0.96) µg/g-creatinine or unnormalized being 0.15 (0.057–0.63) ng/mL. The distribution of urinary cotinine was bimodal (Figure 1). The degree of fitting of the mixture of three normal distributions to the original data evaluated by Kolmogorov-Smirnov test was D = 0.0055 (*p*-value = 0.13).

### 3.3. Cotinine Cut-Off Concentration for Active Smoking

The COV (95% confidential interval) for distinguishing active smokers from others (UCOV) was 36.8 (36.58, 36.84) µg/g-creatinine or 21.5 ng/mL (Figure 1). Of the enrolled participants, 4017 (4.5%) had urinary cotinine concentrations exceeding 36.8 µg/g-creatinine and were active smokers, whereas 3659 (4.1%) had high urinary cotinine concentrations and were current non-smokers (Table 2). By comparison, 82,083 (91.3%) participants had urinary cotinine levels below the COV and were non-smokers, whereas 136 (0.15%) had low urinary cotinine concentrations and were current smokers. Relative to urinary cotinine concentration, the sensitivity of the questionnaire was 0.523 and its specificity was 0.998, with a positive predictive value (PPV) of 0.967 and a negative predictive value (NPV) of 0.957 (Table 2). The COVs slightly changed when using different treatments for data below MRL and different strata of gestational weeks, however, they did not affect sensitivity and specificity (Table S6).

The proportion of participants that had urinary cotinine concentrations exceeding the UCOV in the groups with ETS exposure at a rate of 1 day/week, 2–3 days/week, 4–6 days/week and 7 days/week was 3.4%, 7.6%, 9.4% and 16.0%, respectively (Table S7).

The regression coefficient (standard error) of a single regression model of the number of cigarettes smoked per day and urinary cotinine concentrations was 98 (5.2) µg/g-creatinine, with an adjusted R^2^ value of 0.128 (see Figure S3).

### 3.4. Cotinine Cut-Off Concentration for Passive Smoking

Figure 2 shows a density plot of urinary cotinine concentrations in three groups of pregnant women, i.e., active smokers, passive smokers and non-smokers, as determined by the questionnaire, overlapping with the fitted distribution functions. The COV for discriminating passive smokers from non-smokers (LCOV) was 0.31 (0.308, 0.310) µg/g-creatinine or 0.17 ng/mL. Of the participants, 7734 (9.1%) had urinary cotinine concentrations of 0.31–36.8 µg/g-creatinine and reported exposure to ETS seven times per week, whereas 27,143 (31.9%) had these cotinine concentrations but were unaware of ETS exposure. Relative to urinary cotinine concentrations, the questionnaire had a sensitivity of 0.222, a specificity of 0.977, a PPV of 0.868 and an NPV of 0.644 (Table 3).

## 4. Discussion

This study found that concentrations of urinary cotinine in pregnant women were one or two orders of magnitude lower than those in previous studies [15,27]. The proportion of pregnant women who smoked during pregnancy was similar to the result (5%) from the Japanese national survey conducted in 2010 [28], but was 50% lower than that of previous Japanese epidemiological studies [29,30,31,32]. Using a fitted distribution function, the UCOV was 36.8 µg/g-creatinine. When this COV was considered to represent the true condition, the questionnaire had high PPV (0.967) and NPV (0.957); however, its sensitivity was not satisfactory (0.523). These findings indicate that the answers on the questionnaire to questions about smoking (yes or no) are reliable, but that the questionnaire tends to underestimate the prevalence of smoking among pregnant women. The questionnaire found that 4.6% of pregnant women in JECS smoked, whereas the true smoking rate, based on urinary cotinine levels, was as high as 8%, which was close to the rates of Japanese [29,30,31,32] and American [11] pregnant women. The reason why our questionnaire found a lower proportion of active smokers during pregnancy compared with the previous Japanese studies conducted in 2002–2009 is uncertain; however, it is speculated that it has become more difficult to admit to smoking during pregnancy in recent years.

### 4.1. Urinary Cotinine Concentrations

To the best of our knowledge, this is the first study in Japan to report urinary concentrations of cotinine in pregnant women on this scale. Their median urinary cotinine concentration, 0.24 µg/g-creatinine or 0.15 ng/mL, was one or two orders of magnitude lower than those previously reported in pregnant women, 7.4 ng/mL [15] and 19.7 µg/g-creatinine [27]. However, the maximum urinary cotinine concentration was greater in this study (17,497 µg/g-creatinine) than in a previous study (9776 µg/g-creatinine [27]). We found no correlation between the consumption of foods containing nicotine and urinary cotinine concentrations. The difference in the urinary cotinine concentration was considered to be derived from (1) differences in female smoking rate between countries and (2) differences in the nicotine contents of tobacco that were smoked. Regarding the former point, the smoking rate of Japanese women (11.2%) is lower than those of Spanish and Canadian women (27.4% and 12.0%, respectively) [33]. In terms of the latter point, we did not investigate the brand names of the cigarettes that were smoked. Some reports indicate that Japanese people tend to smoke cigarettes containing less nicotine than Europeans and North Americans [34,35].

### 4.2. Urinary Cotinine Cut-Off Concentrations

The urinary cotinine cut-off concentration distinguishing active smokers from passive smokers and non-smokers in the present study, 36.8 µg/g-creatinine or 21.5 ng/mL, was similar to previously reported concentrations, i.e., 53 µg/g-creatinine or 42, 82 and 200 ng/mL [15,17,36]. To our knowledge, the present study is the second worldwide and the first in Japan to determine the COV based on urinary cotinine levels alone. Further analysis of JECS data should rate smoking status based on urinary cotinine concentrations. Only one other study has reported the relationship between the amount of ETS/cigarette smoking and urinary cotinine concentrations [15]. In that study, 100 ng/mL cotinine was considered a conservative COV for self-reported smokers. Of the participants in our study who reported that they were current non-smokers but were exposed to ETS, 7% had urinary cotinine levels exceeding 100 ng/mL, with these participants regarded as being misclassified.

We found that the distribution of urinary cotinine concentrations was bimodal, with three log-normal distribution functions best fitting the data (l-PDF, m-PDF and h-PDF). The fitted mixture model was not significantly separated from the distribution of the original data (Kolmogorov-Smirnov test, D = 0.0055, *p*-value = 0.13). While the upper mode of the bimodal distribution was assumed to associate with active smoking, no boundary between passive smokers and non-smokers was evident. The EM-like algorithm effectively found two log-normal distributions (l-PDF and m-PDF) in the lower mode. We thus assumed that l-PDF and m-PDF were the distribution functions of non-smokers and passive smokers, respectively. The distributions of non-smokers and active smokers determined by self-reported questionnaires were monomodal, whereas that of passive smokers was bimodal (Figure 2). This implies three possibilities: (1) some participants who said they were passive smokers actually actively smoked, (2) passive smokers had a similar exposure to nicotine as active smokers or (3) a mixture of these two situations. Table S7 indicates that 2675 passive smokers had urinary cotinine concentrations exceeding the UCOV, whereas 3556 (3659 in Table 2 minus 103 participants with missing partners’ smoking status and maternal passive smoking status) participants who had urinary cotinine levels above the UCOV answered ‘did not smoke’ in the questionnaire, leaving 881 (25%) who answered ‘neither active nor passive smoking.’ 

Approximately 43% of the study participants whose urinary cotinine concentrations exceeded 0.31 µg/g-creatinine were unaware of their exposure to ETS. Moreover, 27% of these participants reported that their partners also did not smoke. This indicates that a significant proportion of pregnant women in Japan are unconsciously exposed to tobacco smoke. Urinary cotinine concentrations can be a better marker of smoking status when used in later epidemiological analysis.

### 4.3. Limitations

This study had several limitations. First, the true condition of smoking status was determined based solely on urinary cotinine levels. Nicotine and cotinine have relatively short biological half-lives: 11 h and 17 h to 4 days, respectively [37,38]. Because the samples we collected were spot urine samples, we may have missed appropriate times in participants who smoked infrequently. This may have resulted in an underestimation of smoking prevalence among our participants. In this study, the number of cigarettes smoked did not completely explain the urinary cotinine concentrations. We did not ask for the product names of the cigarettes or how they were smoked in the questionnaire, meaning we could not estimate the nicotine intake. Second, information about nicotine medication was not available in this study. Each cigarette sold in Japan is reported to contain 0.1–1.2 mg of nicotine [39], whereas one sheet of a typical nicotine patch prescribed in Japan contains ~14 mg of nicotine. Thus, nicotine medication may be a significant source of urinary cotinine, resulting in patient misclassification. Third, the food consumption data used in this study were not sufficiently comprehensive to determine the nicotine intake from food items. For example, some foods containing high levels of nicotine such as cauliflower were not taken into consideration because the FFQ did not measure such intake in this study [22,23,37]. These limitations indicate the need to assess other biomarkers of exposure to tobacco smoke, such as 4-(methylnitrosoamino)-1-(3-pyridyl)-1-butanol (NNAL) and some polycyclic aromatic hydrocarbons, in combination with cotinine [38]. It is recommended that multiple biomarkers are used when analysing the associations between environmental exposures, including smoking, during pregnancy and child health outcomes.

## 5. Conclusions

A COV of urinary cotinine concentration, 36.8 µg/g-creatinine, distinguishing active smokers from passive smokers and non-smokers was derived using a fitted distribution function. When this COV was considered to represent the true condition, the sensitivity of a questionnaire addressing maternal smoking was determined to be 0.523, indicating that the proportion of pregnant women in JECS who smoked during pregnancy was underestimated by half. We also found that a large proportion (as high as 78%) of passive smokers might be misclassified as non-smokers.

## Figures and Tables

**Table 1:** Demographic characteristics of the study participants (*n* = 90,049).

Demographic Characteristics	*n* (%)
Maternal age (*n* = 90,033, years, median (range))	31.0 (14.0–50.0)
Gestational week (*n* = 90,049, weeks, median (range))	27.0 (15.0–41.0)
Household income (*n* = 83,755)	
<4 million yen (~37,000 USD)	27,694 (33.1)
4–6 million yen	33,781 (40.3)
>6 million yen (~55,000 USD)	22,280 (26.6)
Maternal smoking status (*n* = 89,895)	
No	85,742 (95.4)
Yes	4153 (4.6)
Partner smoking status (*n* = 89,463)	
No	47,611 (53.2)
Yes	41,852 (46.8)
Passive smoking (*n* = 89,788)	
None	55,859 (62.2)
One day per week	10,647 (11.9)
Two to three days per week	7404 (8.2)
Four to six days per week	4372 (4.9)
Seven days per week	11,506 (12.8)
Number of cigarettes smoked per day(*n* = 4114, cigarettes per day, median (range))	10 (0–60)
Urinary cotinine concentration	
(µg/g-creatinine, median interquartile range (IQR))	0.24 (0.083–0.96)
(ng/mL, median (IQR))	0.15 (0.057–0.63)
Urinary creatinine concentration (mg/dl, median (IQR))	74.3 (42.3–116.4)

**Table 2:** Accuracy of the questionnaire on active smoking using the urinary cotinine cut-off concentration of 36.8 µg/g-creatinine (*n* = 89,895).

		True Condition	
		≥36.8 µg/g-Creatinine	<36.8 µg/g-Creatinine
Questionnaire (Smoking)	Yes	4017	136
	No	3659	82,083
Sensitivity		0.523	
Specificity		0.998	
Positive Predictive Value		0.967	
Negative Predictive Value		0.957	

**Table 3:** Accuracy of the questionnaire on passive smoking using the urinary cotinine cut-off concentration of 0.31 µg/g-creatinine (*n* = 85,103).

		Partner Smoking		Passive Smoking 7 Days		≥4 Days		≥2 Days		≥1 Day	
		≥COV	<COV	≥COV	<COV	≥COV	<COV	≥COV	<COV	≥COV	<COV
Questionnaire Response	Yes	23,464	14,602	7734	1178	10,605	2262	15,067	4687	19,797	10,294
	No	11,413	35,624	27,143	49,048	24,272	47,964	19,810	45,539	15,080	39,932
Sensitivity		0.673		0.222		0.304		0.432		0.568	
Specificity		0.709		0.977		0.955		0.907		0.795	
Positive Predictive Value		0.616		0.868		0.824		0.763		0.658	
Negative Predictive Value		0.757		0.644		0.664		0.697		0.726	

## Supplementary Materials

The following are available online at https://www.mdpi.com/1660-4601/17/15/5537/s1. Table S1: LC gradient conditions; Table S2: LC conditions; Table S3: Monitoring of ion mass transition; Table S4: Ion source and collision cell conditions; Table S5: Range of calibration curves for cotinine; Table S6: UCOV, LCOV and sensitivity and specificity of UCOV in four different datasets; Table S7: The proportion of participants with urinary cotinine concentrations exceeding the UCOV in each ETS exposure group; Figure S1: Flow chart of the study participants; Figure S2: Correlation between the consumption of potential food sources of nicotine and urinary cotinine concentrations among non-smokers (*n* = 29,908, Spearman’s rank correlation r_s_ = 0.046); Figure S3: Single regression analysis showing the relationship between the number of cigarettes smoked per day and urinary cotinine concentrations of current smokers (adjusted R^2^ = 0.128); Figure S4: Sample treatment for measurement of urinary cotinine.

## Appendix A. Urinary Cotinine Analysis Method

### Appendix A.1. Chemicals and Reagents

All reagents were of high-quality grade unless specified otherwise. Water was brought to TOC ≤ 15 ppb using a Milli-Q Integral 5 or 10 and EQP-10 L system (Merck Millipore, Bedford, MA, USA). Acetic acid (99.8% purity), 28% w/v ammonia solution, formic acid and acetone (99.0% + purity) were purchased from FUJIFILM Wako Pure Chemical Corporation (Osaka, Japan). Methanol (MeOH; 99.8% purity) was purchased from Nacalai Tesque, Inc. (Kyoto, Japan). A standard solution of cotinine (500 ng/mL), as well as an internal standard (IS) solution containing stable isotope-labelled cotinine (^13^C_3_), were purchased from Cambridge Isotope Laboratories, Inc. (Andover, MA, USA). All glassware was precleaned with acetone immediately before measurements.

### Appendix A.2. Sample Preparation

Ten microliters of IS solution, containing 3 ng/mL of ^13^C_3_-cotinine, and 400 µL of 1.4% ammonia solution were added to each 100 µL aliquot of urine sample. The sample was vortex mixed and loaded onto an Oasis MAX (mixed-mode, strong anion exchange) 96-well plate, containing 30 mg sorbent per well and a particle size of 30 µm (Waters Corp., Milford, MA, USA), preconditioned with 500 µL of MeOH followed by 500 µL of 1.4% ammonia. The cartridge was washed with 500 µL of 1.4% ammonia, and the target compound was eluted with 200 µL of 50% (v/v) MeOH. This eluate was dissolved in 300 µL of water and mixed at 300 rpm for 10 min. A 10 µL aliquot of each was injected into a high-performance liquid chromatography–tandem mass spectrometer (LC–MS/MS) (see Figure S4).

### Appendix A.3. Instrument Analysis and Calculations

The LC (Nexera X2 system, Shimadzu, Corporation, Kyoto, Japan) and MS/MS (Triple Quad 6500, AB Sciex Pte. Ltd., Framingham, MA, USA) systems were operated using electrospray ionization (ESI) positive in the multiple reaction monitoring (MRM) mode. The analytical column was a CAPCELL CORE ADME, 2.1 mm I.D. × 50 mm, 2.7 µm column (Osaka Soda Co., Ltd., Osaka, Japan). The column flow rate was 0.4 mL/min, and its temperature was 40 °C. The typical routine operating conditions and data acquisition settings are documented, and the calibration range is shown in Tables S1–S5. All samples outside the calibration range were reanalysed following further dilution.

The quality control (QC) sample, consisting of Milli-Q water containing 0.2 ng/mL cotinine, was treated with the same procedure as the urine samples, with four or more replicates analysed in each analytical sequence. MRL was calculated based on the lowest concentration MRL (LCMRL) using QC samples, as described [40].

### Appendix A.4. Quality Control

The ten-point calibration curve had a coefficient of determination (R^2^) higher than 0.992. Repeatability and intermediate precision were determined based on ISO 5725:1994 and 27148:2010, with QC sample measurements (*n* = 367) used for calculations. QC for day-to-day analysis was determined using a Shewhart control chart ($\bar{X}$-Rm control chart) according to ISO 7870. The percent recovery of target compounds was calculated by fortifying a pooled urine sample with known concentrations of cotinine standards and measuring these concentrations in seven replicates, resulting in 106% recovery of cotinine. The standard reference material, SRM 3673 (Organic Contaminants in Non-Smokers’ Urine, National Institute of Standards and Technology), was also analysed in seven replicates to determine the accuracy of the measurement. Mean ± SD urinary cotinine concentration in the SRM was 23.3 ± 0.3 ng/mL (relative standard deviation, 1.4%), in agreement with the reference value (24 ± 1 ng/mL). Following sample transfer to the contract laboratory, urinary creatinine concentration and specific gravity were analysed using an enzymatic assay and refraction method, respectively.
