# Supplementary material for: Determination of Urinary Cotinine Cut-Off Concentrations for Pregnant Women in the Japan Environment and Children’s Study (JECS)
Source: Int J Environ Res Public Health. 2020 Jul 31;17(15):5537. doi: 10.3390/ijerph17155537 (PMC7432074; doi:10.3390/ijerph17155537)
Supplement: Supplementary file 1 [file ijerph-17-05537-s001.pdf]

Table S1. LC gradient conditions.

Time (min)	LC Pump	
	**% Pump-1 0.1% Acetic acid**	**% Pump-2 Methanol**
0.00	98	2
0.50	98	2
3.00	75	25
3.50	75	25
3.51	20	80
4.00	20	80
4.01	98	2
6.50	98	2

Table S2. LC conditions.

**LC parameters**	
Pump-1	0.1% Acetic acid
Pump-2	Methanol
Flow rate	*0.4 mL/min*
Injection volume	*10 µL*
Column temperature	*40°C*

Table S3. Monitoring of ion mass transition.

	**Target ion (m/z)**		**Qualifier ion (m/z)**		**Declustering Potential (V)**		**Collision Energy (eV)**		**Ionization mode**
	**Precusor**	**Product**	**Precusor**	**Product**	**Target ion**	**Qualifier ion**	**Target ion**	**Qualifier ion**	
Native	177.0	80.0	177.0	98.0	75.0	75.0	28.0	27.0	positive
Internal standard ($^{13}C_3$)	180.0	80.1	–	–	61.0	–	30.0	–	positive

Table S4. Ion source and collision cell conditions.

Parameters	Setting
IonSpray voltage (V)	*5000*
Heating gas temperature (℃)	*650*
Nebulizer gas (psi)	*50*
Heating gas (psi)	*80*
Curtain gas flow (psi)	*30*
Collision gas pressure	*10*

Table S5. Range of calibration curves for cotinine.

Native	Concentrations (ng/mL)											Internal standard	Concentrations (ng/mL)
Cotinine	0	0.006	0.01	0.02	0.06	0.1	0.2	0.4	0.6	1	2	*Cotinine*-$^{13}C_3$	0.06

Table S6. UCOV, LCOV and sensitivity and specificity of UCOV in four different datasets.

Operation	N	UCOV, LCOV	Sensitivity, specificity of UCOV
Current	89,895	36.7, 0.31	0.523, 0.998
Data <MRL substituted by half the MRL	89,895	41.5, 0.34	0.526, 0.998
Excluding data <MRL	80,023	32.9, 0.36	0.521, 0.998
Gestational week <26.9	41,310	34.6, 0.30	0.526, 0.999
Gestation week ≥26.9	48,555	38.1, 0.31	0.519, 0.998

UCOV; cut-off value for dividing active smokers from others, LCOV; cut-off value for discriminating passive smokers from non-smokers, MRL; minimum reporting level.

Table S7. The proportion of participants with urinary cotinine concentrations exceeding the UCOV in each ETS exposure group.

		Number of participants (%)	
ETS exposure	N	<UCOV	UCOV≤
1 day/week	10,337	9,990	347 (3.4%)
2-3 days/week	6,887	6,361	526 (7.6%)
4-6 days/week	3,955	3,582	373 (9.4%)
7 days/week	8,912	7,483	1,429 (16.0%)

UCOV; cut-off value for dividing active smokers from others, ETS; environmental tobacco smoke.

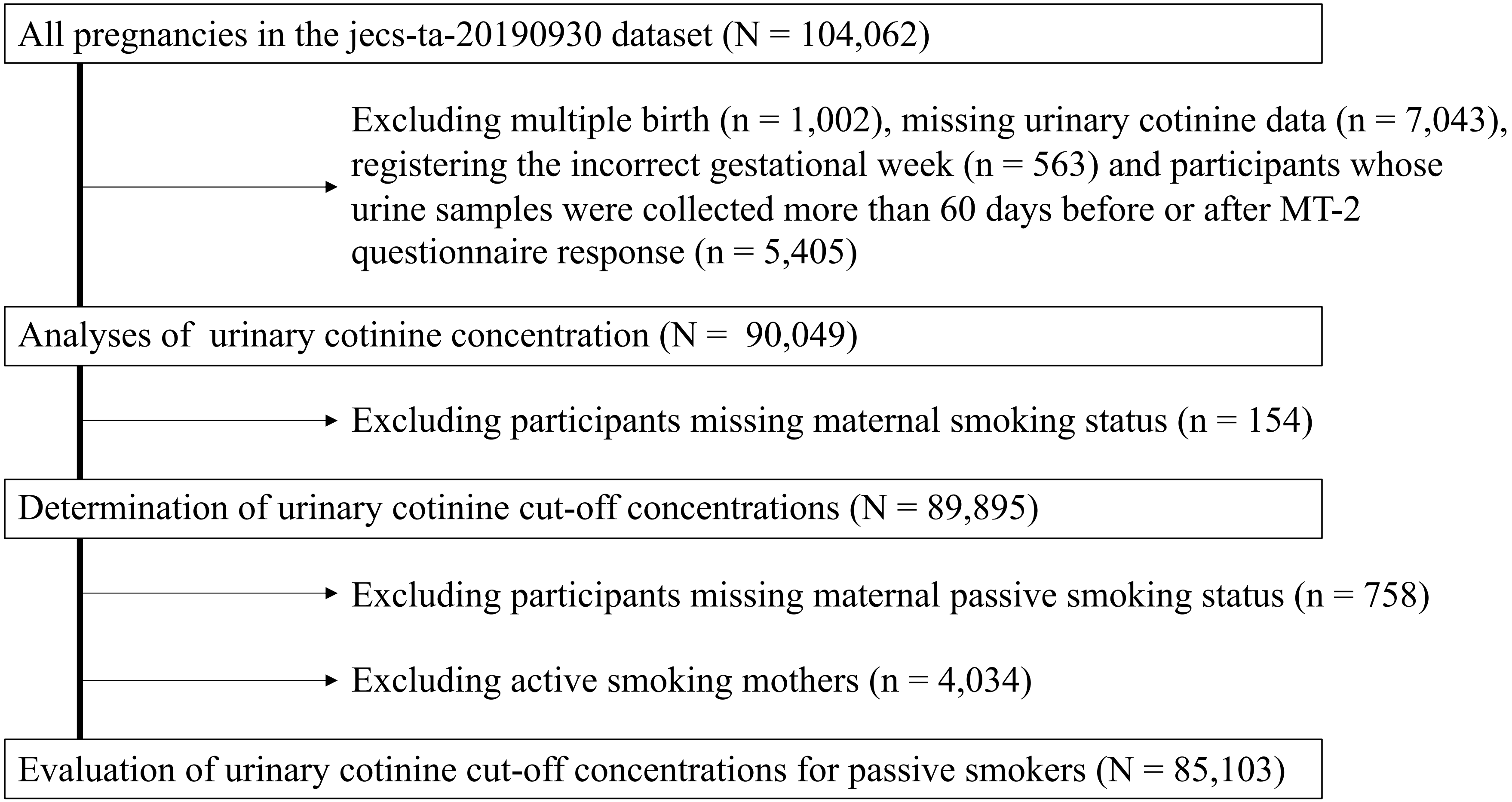

Figure S1. Flow chart of the study participants.

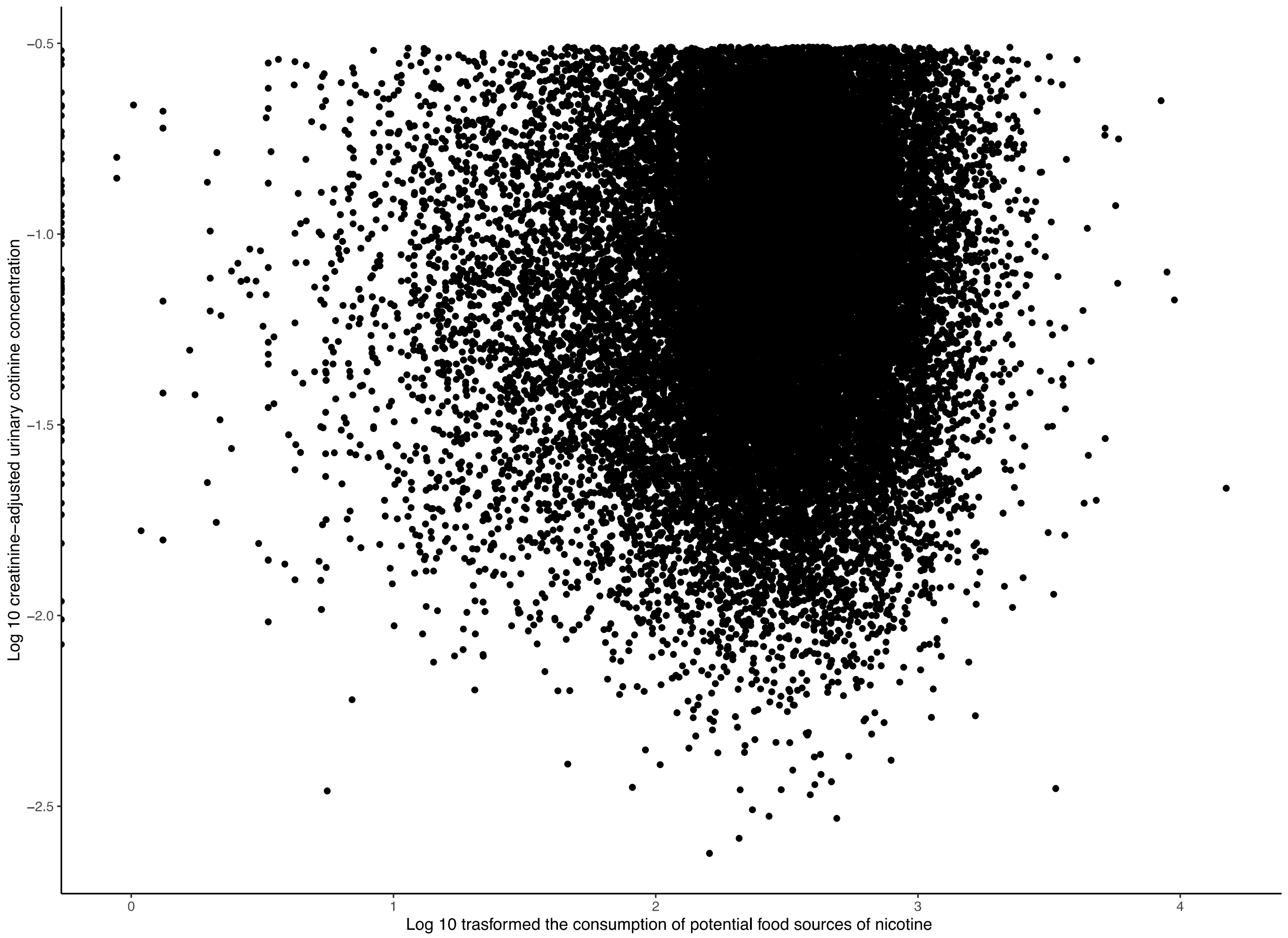

Figure S2. Correlation between the consumption of potential food sources of nicotine and urinary cotinine concentrations among non-smokers (N = 29,908, Spearman's rank correlation r_s = 0.046).

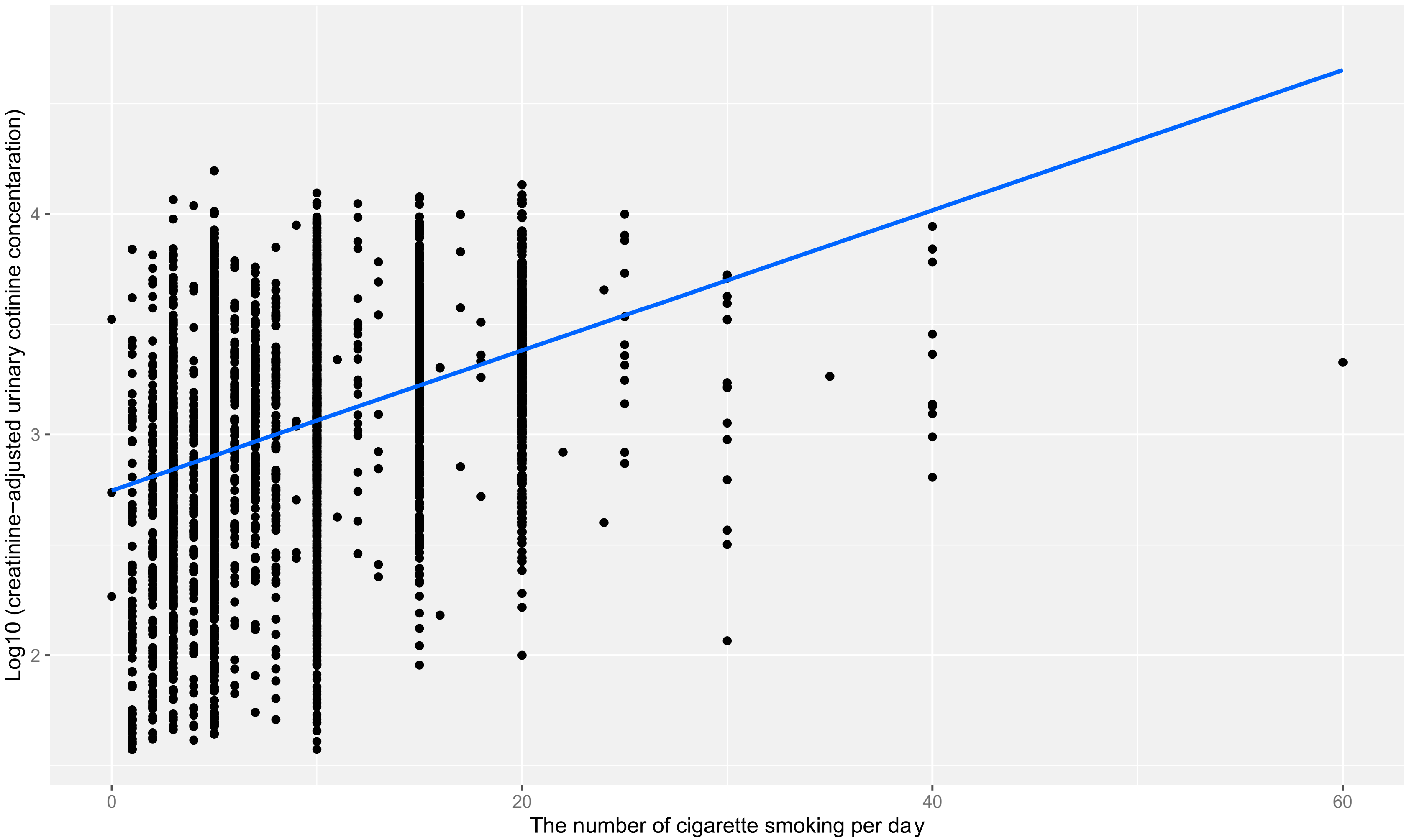

.

Figure S3. Single regression analysis showing the relationship between the number of cigarettes smoked per day and urinary cotinine concentrations of current smokers (adjusted $R^2 = 0.128$).

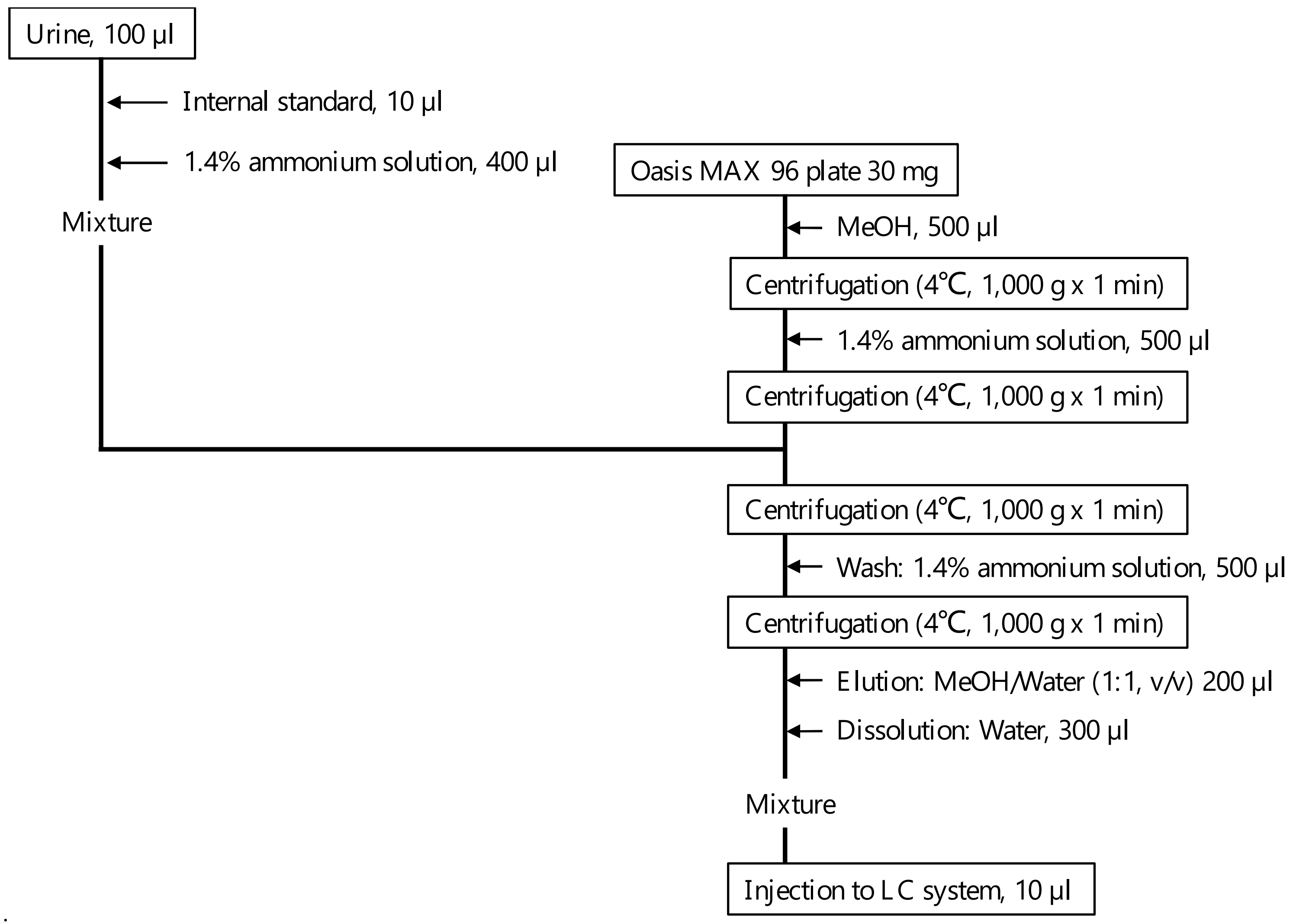

Figure S4. Sample treatment for measurement of urinary cotinine.
